# Lessons learned from people-centred TB care and support interventions in the Russian Federation

**DOI:** 10.5588/ijtldopen.25.0844

**Published:** 2026-06-15

**Authors:** N. Stavitskaya, I. Felker, E. Nemkova, M. Vujnovic, B. Berdyklychev, S. Yegeubayeva

**Affiliations:** 1 Novosibirsk Tuberculosis Research Institute, WHO Collaborating Centre, Novosibirsk, Russia;; 2 Novosibirsk State Medical University, Novosibirsk, Russia;; 3 Independent Public Health Researcher, Belgrade, Serbia;; 4 WHO Country Office in the Russian Federation, Moscow, Russia.

**Keywords:** tuberculosis, treatment support, MDR-TB, Siberia, Far Eastern federal districts

## Abstract

**BACKGROUND:**

In recent years, key TB indicators have improved in the Siberian and Far Eastern federal districts (FDs) of Russia, but these regions still face the challenge of reducing the numbers of patients with drug-resistant-TB and TB/HIV co-infection. TB care and support interventions recommended by the WHO have been implemented, such as home-based and outpatient treatment, use of digital technologies and provision of psychosocial support.

**METHODS:**

The study used a short survey to assess the influence of these interventions on patient adherence to treatment during the period 2018–2022 in the Siberian and Far Eastern FDs. The survey was sent to the heads of TB clinics in the 21 regions.

**RESULTS:**

Of the 21 regions invited to take part in the study, 20 returned answers; 14 of these completed the survey fully, and the other six only partially (as some of the data requested are not routinely collected in those regions). The results show that TB care and support interventions, especially psychosocial interventions, are associated with a reduction of number of patients lost to follow-up and the number of TB cases.

**CONCLUSION:**

Care and support interventions have only been implemented for a relatively short period, and their use needs to be continued, along with the creation of a data repository to enable monitoring and further studies to meet targets on TB control.

Between 2015 and 2020, the WHO European Region achieved a 25% reduction in TB incidence and a 26% reduction in mortality.^1^ However, even though the region has only 3% of the global-diagnosed TB cases recorded, it has 24% of the global-diagnosed cases of multidrug-resistant or rifampicin-resistant TB (MDR/RR-TB) and 47% of the cases of pre-extensively drug-resistant TB.^2^ In the same period, TB incidence in the Russian Federation decreased by 46.1% and TB mortality decreased by 58.7%,^3–5^ trends that were also seen in the Siberian and Far Eastern federal districts (FDs) of the Russian Federation, where TB incidence decreased by 45.5% and 50.7%, respectively, in the period 2015–2022. By 2022, the proportion of bacteriologically confirmed cases of MDR-TB among the total number of patients with active TB had decreased but remained at high levels: 60% in the Siberian and 58.8% in the Far Eastern FDs.^4–6^

The high incidence of MDR/RR-TB means that the commitment to reaching the WHO European Region milestone of an 80% treatment success rate for MDR/RR-TB by 2025, in comparison with 2015 levels, is a national priority. The Siberian and the Far East FDs comprise 21 regions, which differ significantly in geography, population demographics and TB epidemiology. These regions constitute 66.1% of the Russian Federation territory but are home to only 16.8% of the total population.^7^ Some regions have implemented patient-centred care for TB patients and use the WHO-recommended TB care and support interventions (TBCSIs), including using digital technologies (DTs) to support treatment adherence.^8–12^ The COVID-19 pandemic and the resultant movement-restriction measures accelerated the introduction of home-based treatment and led to an increase in the use of DT, including video-supported treatment (VST), call centres and tracers.

This study analyzes the influence of people-centred TBCSIs on adherence to treatment in the Siberian and the Far Eastern FDs during 2018–2022.

## METHODS

The study used a short survey to analyse the influence of people-centred TBCSIs on patient adherence in the Siberian and the Far Eastern FDs during 2018–2022. This period was chosen to reflect the changes in TB care provision that may have occurred in connection with the COVID-19 pandemic. Information on the five interventions used to treat TB in the Siberian and the Far Eastern FDs such as daily visits to hospital TB clinics (‘day hospital’), home-based treatment, outpatient (ambulatory) care, DT (VST, text messages to mobile devices, telephone calls) and psychosocial support models was collected. The list of questions was sent to TB clinics, and data were requested on the numbers of patients treated with any of the five interventions and the number of patients lost to follow-up (PLTFU) during 2018–2022. The heads of the TB clinics in the 21 regions were also asked to share their expert opinions on the effectiveness of the services implemented. Of the 21 regions invited to take part in the study, 20 regions in the Siberian and the Far Eastern FDs returned their answers; 14 regions completed fully, and the other six partially completed as some of the data requested are not routinely collected in those regions.

The median, Spearman correlation coefficient and line regression were used for analysis. The number of patients treated using day hospital, home-based treatment, outpatient care, DTs and psychosocial support models were defined as the independent variables. The number of TB cases and PLTFU in the regions were defined as dependent variables.

### Ethical statement

The study was conducted retrospectively, collecting only aggregated data. No personal patient data were included in the questionnaire. Therefore, ethics committee approval was not required.

## RESULTS

The most widely used model for TB care in the Russian Federation is day hospital visits. This model is used in 18 of the 20 regions. Some regions have used this model of care for the last 20 years and others for less than 10 years. However, the coverage of patients was limited and only six regions treated over 30% of all TB patients in this way.

In 2018–2022, only 9 out of the 20 participating regions used home-based treatment. Three regions have been using this model for 20 years or longer. Overall, the proportion of patients treated using home-based TB treatment ranged from 5% to 35% in the different regions. Two regions did not use any hospital-replacement models for TB care.

DTs are widely used in health care. Although, DTs were used before 2020, in some regions, the introduction of VST accelerated during the pandemic and its implementation was directly related to the movement restrictions. Various mobile phone applications for VST were used in 13 out of the 20 regions. Another three regions implemented call centres that reminded patients to take their medications via individual telephone calls and text messages. Four regions did not use DTs at all. Although the number of regions introducing DTs for TB care and support has increased in recent years, the actual intensity of usage has remained low. In most regions, fewer than 20% of all treated patients used DTs, and only three regions had an average of 40% patient coverage.

Psychosocial support was provided in 13 regions, with half of these regions providing psychosocial support coverage to 20%–60% of patients, and the other half providing less than 5% coverage.

Nine regions provided data on the proportion of patients treated on a fully outpatient basis. In 2018–2022, this indicator was less than 10%, and in five regions this proportion did not ever exceed 5%. Only two regions reached 30% coverage with hospital-replacement models of TB care. Due to the COVID-19 pandemic, there was a slight increase in the proportion of patients receiving outpatient treatment in nine regions in 2020, but, by 2022, four of these regions had returned to the proportions seen in 2018, or lower.

In 2018–2019, the proportion of PLTFU did not exceed 10.5% among all treated patients with active TB. In 2020, the proportion of PLTFU was 11%, an increase which is most likely due to the COVID-19 restrictions. The subsequent rapid implementation of DTs helped to reduce the number of PLTFU to 8.9% in 2022. In 16 of the regions, the proportion of PLTFU in patients treated with TBCSIs was significantly lower, at 2.4%–3.0%; four regions did not respond to this question.

The heads of 12 TB clinics all thought that the introduction of TBCSIs contributed to better adherence to treatment and reported that the number of patients interrupting treatment decreased, especially when psychosocial support, VST and other DTs were used. Two of the 20 regions reported that they had not seen any impact of the interventions on the effectiveness and success of TB treatment. One third of the heads of clinics did not respond to the question.

An analysis of the dependence of the level of TB cases and the frequency of use of TBCSIs showed that regions using more interventions had fewer TB cases (Table 1). Across all responded regions day hospital was the most popular model with 33.9% of use, with psychosocial support the second most popular, at 28.4%. Home-based treatment had 12.2% of use; DTs, 11.2%; and outpatient treatment, 10.03%.

**Table 1.** Use of TB care and support interventions and active TB cases per 100,000 population per year, across the 21 regions of the Siberian and Far Eastern regions of the Russian Federation, 2018–2022.

TB care and support intervention	Active TB cases per 100,000 population in the regions per year lower than the median		Median (per 100,000 population)	Active TB cases per 100,000 population in the regions per year higher than the median	
	%	SD^A^		%	SD
Home-based treatment	13.2	0.106	131.9	8.3	0.097
Day hospital	42.5	0.276		17.9	0.118
Digital technologies	14.0	0.150		8.0	0.088
Psychosocial support	32.4	0.297		22.9	0.218
Outpatient treatment	11.7	0.097		7.3	0.094

A SD = standard deviation

All five interventions are negatively correlated with the number of active TB cases per 100,000 population in the regions per year indicating that the more frequent use of TBCSIs relates to a lower number of active TB cases per region per year (Table 2).

**Table 2.** Correlation between use of TB care and support interventions and active TB cases per 100,000 population per year, across the 21 regions of the Siberian and Far Eastern regions of the Russian Federation, 2018–2022.

	Home-based treatment		Day hospital		Digital technologies		Psychosocial support		Outpatient treatment	
TB cases per 100,000 population in the regions, per year	r*	P**	r	*P*	r	*P*	r	*P*	R	*P*
	−0.474	0.017	−0.631	0.000	−0.395	0.002	−0.271	0.010	−0.342	0.002

*r = Spearman correlation.

**P = *P*-value.

Two regression models of the multivariate interactions were created: TBCSIs and active TB cases per 100,000 population in the regions per year; and TBCSIs and the number of PLTFU. In the TBCSIs and active TB cases per 100,000 population in the regions per year regression model, psychosocial support was negatively correlated with the rate of active TB cases per 100,000 population in the regions per year (β = −0.206, *P* = 0.051). In the TBCSIs and number of PLTFU regression model, day hospitals was correlated with higher number of PLTFU (β = 0.426, *P* = 0.013), and psychosocial support was correlated to lower numbers of PLTFU (β = −0.332, *P* = 0.050).

## DISCUSSION

During 2018–2022, in the Siberia and Far Eastern FDs, TBCSIs were scaled up, with 20 of 21 regions using one or more interventions to reduce the number of TB cases and the number of PLTFU. The day hospital visits provision model has been used for a long time and is the most popular TB service model in the regions that took part in the study, but the analysis showed that it was a predictor of higher numbers of PLTFU. One reason for the higher number of PLTFU could be the difficulties faced by patients in organising day hospital visits, including daily transport expenses to hospitals.

Home-based TB treatment and full outpatient treatment are models that are widely used in other countries, but which are less popular in Russia, and their use remains low in the studied regions. One of the reasons is the recommendation that patients with MDR-TB and HIV co-infection need to be hospital inpatients under continuous supervision from a doctor, and patients with MDR-TB/HIV co-infection make up a significant proportion of the TB patients in the regions studied. However, studies from South Africa and Uganda have reported the successful use of the hospital-at-home model for patients with MDR-TB/HIV co-infection^13,14^; thus, introducing these models, and monitoring their implementation to gather evidence on their effectiveness, is needed to further scale up the use of home-based treatments.

The COVID-19 pandemic movement-restriction measures contributed to the accelerated introduction of DTs for TB care. The number of regions using DTs in 2020–2021 increased threefold. This study could not demonstrate that the introduction of DTs reduces the number of PLTFU, perhaps because the proportion of patients covered by VST in most regions did not exceed 20%. However, the study showed that the regions with higher use of DTs did have lower levels of TB cases. A significant increase in TB treatment adherence and effectiveness has been observed when DTs, such as VST and text message reminders, have been used.^15–19^ In the Irkutsk region in the Russian Federation, increased adherence to TB treatment and antiretroviral treatment in patients with TB/HIV co-infection was seen in patients who used a mobile phone application to receive treatment reminders.^20^ However, a study in China did not showed any effect on the use of digital adherence technology interventions on treatment outcomes, although treatment adherence was increased in the intervention group compared with the control group.^21^

Psychosocial support plays a significant role in increasing treatment adherence, especially for patients with MDR-TB/HIV co-infection.^22–25^ In this study, the presence of psychosocial support in the regression model was a predictor for reduced treatment interruption and a lower rate of active TB cases, as has been reported in studies in other countries.

The studied regions have a high burden of TB, MDR-TB and HIV co-infection, and a lot of effort has been invested in implementing TBCSIs to increase adherence to treatment in patients with MDR-TB, to contribute to achieving the targets on treatment success rate and number of TB cases defined in the national priorities of the Russian Federation and to align with WHO priorities to End TB in the European Region. Patient-centred care requires not only the availability of day hospitals but also the provision of home-based treatment and directly observed treatment locations situated as close as possible to patients’ homes. Increasing the use of DTs and psychosocial support to ensure adherence to treatment will help to reduce the number of PLTFU and increase the effectiveness and success of TB treatment in the Siberian and Far Eastern FDs.

Any direct correlation between the interventions and a positive effect on the numbers of people with active TB, or the numbers of PLTFU, is uncertain as the intervention coverage is relatively small. The minimal data set that needs to be collected to monitor the performance of TBCSIs and to analyse the impact on treatment adherence needs to be defined.

Not all the regions included in the study were able to provide complete data for the study period as most of the information being requesting is not routinely collected. Some models, such as day hospital visits, have been implemented for a long time, but others, such as home-based treatment and DTs, were started 2–3 years ago, and others were introduced during the COVID-19 pandemic and have since been removed from routine services. The lack of data made it impossible to observe some expected correlations between the selected variables and the strong relationships between the indicators and predictors to find differences in the provision of TB care that has occurred in connection with the pandemic.

The study was unable to analyse treatment success rate in the regions due to a lack of routine data on the use of WHO-recommended^13,26^ shorter, fully oral MDR-TB therapy. The Ministry of Health of the Russian Federation approved updated federal clinical TB guidelines several times, with the most recent approval in 2026, which permits the introduction of shortened, all-oral regimens for treating patients with MDR-TB into routine practice,^27^ after the country conducted pilot studies to evaluate the effectiveness of these TB chemotherapy regimens in various local conditions.

Further studies will contribute to gathering the evidence needed for the introduction of these practices at the field level.
